# TSC1 Controls Distribution of Actin Fibers through Its Effect on Function of Rho Family of Small GTPases and Regulates Cell Migration and Polarity

**DOI:** 10.1371/journal.pone.0054503

**Published:** 2013-01-23

**Authors:** Maki Ohsawa, Toshiyuki Kobayashi, Hidehiro Okura, Takashi Igarashi, Masashi Mizuguchi, Okio Hino

**Affiliations:** 1 Department of Pediatrics, Graduate School of Medicine, The University of Tokyo, Tokyo, Japan; 2 Department of Pathology and Oncology, Juntendo University School of Medicine, Tokyo, Japan; 3 Department of Neurosurgery, Juntendo University School of Medicine, Tokyo, Japan; 4 Department of Developmental Medical Sciences, Graduate School of Medicine, The University of Tokyo, Tokyo, Japan; Beatson Institute for Cancer Research Glasgow, United Kingdom

## Abstract

The tumor-suppressor genes *TSC1* and *TSC2* are mutated in tuberous sclerosis, an autosomal dominant multisystem disorder. The gene products of *TSC1* and *TSC2* form a protein complex that inhibits the signaling of the mammalian target of rapamycin complex1 (mTORC1) pathway. mTORC1 is a crucial molecule in the regulation of cell growth, proliferation and survival. When the TSC1/TSC2 complex is not functional, uncontrolled mTORC1 activity accelerates the cell cycle and triggers tumorigenesis. Recent studies have suggested that TSC1 and TSC2 also regulate the activities of Rac1 and Rho, members of the Rho family of small GTPases, and thereby influence the ensuing actin cytoskeletal organization at focal adhesions. However, how TSC1 contributes to the establishment of cell polarity is not well understood. Here, the relationship between TSC1 and the formation of the actin cytoskeleton was analyzed in stable TSC1-expressing cell lines originally established from a *Tsc1*-deficient mouse renal tumor cell line. Our analyses showed that cell proliferation and migration were suppressed when TSC1 was expressed. Rac1 activity in these cells was also decreased as was formation of lamellipodia and filopodia. Furthermore, the number of basal actin stress fibers was reduced; by contrast, apical actin fibers, originating at the level of the tight junction formed a network in TSC1-expressing cells. Treatment with Rho-kinase (ROCK) inhibitor diminished the number of apical actin fibers, but rapamycin had no effect. Thus, the actin fibers were regulated by the Rho-ROCK pathway independently of mTOR. In addition, apical actin fibers appeared in TSC1-deficient cells after inhibition of Rac1 activity. These results suggest that TSC1 regulates cell polarity-associated formation of actin fibers through the spatial regulation of Rho family of small GTPases.

## Introduction

Tuberous sclerosis complex (TSC) is an autosomal dominant neurocutaneous syndrome caused by mutation of either *TSC1* or *TSC2*, tumor suppressor genes that encode the proteins TSC1 (hamartin) and TSC2 (tuberin), respectively [1]. TSC is characterized by the occurrence of benign tumors, or hamartomas, involving multiple organ systems including the central nervous system [2]. In addition, most TSC patients show neurological symptoms, such as seizures, mental retardation and autism [3].

TSC1 and TSC2 form a complex that negatively regulates the activity of mammalian target of rapamycin (mTOR) by inhibiting Ras homolog enriched in brain (Rheb). The active form of Rheb enhances phosphorylation of the mTOR substrates p70 S6 kinase (S6K) and 4E-BP1, and promotes cell growth and proliferation [4], [5]. Upstream of TSC1/2, Akt can act as an inhibitor of TSC1/2 [6]. Furthermore, several studies have shown that loss of TSC1/2 complex function results in a decrease in Akt phosphorylation and activation [7], [8].

Although no clear-cut genotype-phenotype correlations have been established, patients with *TSC1* mutations tend to be less severely affected than those with *TSC2* mutations [9], [10]. Evidence that TSC1 and TSC2 can also act separately is accumulating. Thus, TSC2 has been reported to possess GTPase-accelerating protein (GAP) activity for Rheb [11], to harbor transcriptional activation domains [12] and to modulate transcription by members of the steroid receptor superfamily of genes [13]. TSC2 has also been suggested to regulate neuronal differentiation [14] and to determine polycystin-1 functional localization [15]. In contrast to the increased understanding of the functions of TSC2, much less is known about TSC1. Two studies have suggested that TSC1 is needed for the GAP activity of TSC2 [16], [17]; however, another study reported that TSC1 has no effect on TSC2-associated GAP activity [18]. GSK3 has been shown to phosphorylate TSC1 [19]; this phosphorylation increases the stability of the TSC1/TSC2 complex and attenuates β-catenin signaling. TSC1 can interact with members of the ezrin-radixin-moesin family [20] and with neurofilament-L [21], suggesting that it may play a role in the actin cytoskeleton organization. However, our understanding of the role of TSC1 is still limited and further evaluation of its primary function is needed.

The Rho family of small GTPases (Rho, Rac and Cdc42) are molecular switches that cycle between active (GTP-bound) and inactive (GDP-bound) states. When activated, the Rho family of GTPases undergoes a conformational change, enabling the recruitment of effector proteins that mediate downstream effects. Rho GTPases have effects on the actin cytoskeleton, and play a role in cell polarity, migration and proliferation [22].

At the front of a migrating cell, actin assembly drives the extension of flat membrane protrusions called lamellipodia, and finger like protrusions called filopodia [23]. At the leading edge of each lamellipodium, the cell forms adhesions that connect the extracellular matrix to the actin cytoskeleton to anchor the protrusion and pull the cell body. Finally, to move forward, the cell retracts its trailing edge by combining actomyosin contractility and disassembly of adhesions at the rear. Rho GTPases control all these aspects. Rac1 regulates actin polymerization at the front to induce the formation of membrane ruffles and lamellipodia. Cdc42 acts at the front to induce filopodia formation. RhoA promotes the formation of stress fibers that are linked to focal adhesions [22], [24].

Adhesion between adjacent cells and between cells and extracellular matrix is critical to tissue morphogenesis. The apical surface of the epithelium is exposed to the environment or to the lumen, whereas the basal surface faces the extracellular matrix. There are five different types of junctions [25], [26], [27], and three of them are connected to the actin cytoskeleton. The first of these three are tight junctions (TJs) that form the most apical component of the junctional complex, seal adjacent cells together, and function as selective permeability barriers. Claudins and occludins, two of the TJ transmembrane proteins so-far identified, are linked to the actin cytoskeleton through zona occludens (ZO) proteins. The second type is adherens junctions (AJs), which are positioned immediately below TJs, and are formed by classical cadherins that are linked to the actin cytoskeleton through adaptor proteins, such as catenins and vinculin. The third type is focal adhesions, which are cell–matrix junctions that are formed by integrins. Focal adhesions are composed of over 125 protein species, including adaptor proteins, such as talin and vinculin, and link to actin filaments and scaffold proteins such as paxillin. Rho GTPases have a role in cell-cell junction formation. RhoA is required for junction dynamics. Rac1 and Cdc42 have also been linked to actin cytoskeleton, cell polarity and paracellular permeability regulation relevant to TJs [28].

Very little is known about the function of TSC proteins in the regulation of Rho family of GTPases and actin cytoskeleton. As mentioned above, TSC1 can bind to the ezrin-radixin-moesin (ERM) family of actin-binding proteins and can regulate cell adhesion via the Rho-mediated signaling pathway [20]. Furthermore, it has also been documented that TSC1 can inhibit Rac1, while TSC2 can counteract this inhibition [29]. These observations suggest the importance of TSC1 and TSC2 for the regulation of cell adhesion through the Rho family of small GTPases. However, the function of TSC1 in actin fiber remodeling, other than for focal adhesion, is not clear.

In this study, we explore the role of TSC1 in actin cytoskeleton organization in a novel mouse renal tumor cell line. We show that TSC1 regulates the actin fiber remodeling associated with apical-basal cell polarity. In addition to inhibiting Rac1 activity, TSC1 may also control the spatial regulation of RhoA activity, and thereby regulate cell polarity. Our findings suggest that TSC1 regulates the actin cytoskeleton via a novel pathway in mouse renal tumor cells.

## Results

### TSC1 Inhibits Proliferation of *Tsc1*-deficient Cells

**Figure 1:**
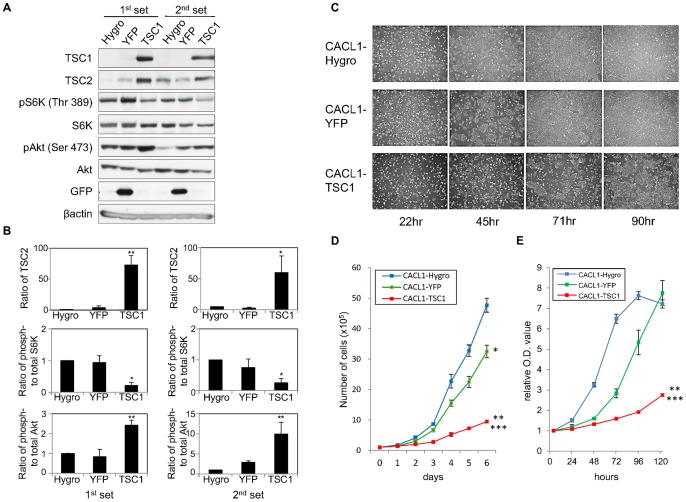
TSC1 inhibits proliferation of TSC1-deficient cells. (A) Restoration of TSC1 suppresses the mTOR signaling pathway in TSC1-deficient renal tumor cells. Total cell lysates were analyzed by immunoblotting with the indicated antibodies. Two independent sets of each cell line were examined. (B) Results from three independent experiments as for panel A were quantified. Relative TSC2 protein levels (top), the ratio of phospho-S6K to total-S6K (middle) and the ratio of phospho-Akt (bottom) are shown as means ± SE. Significant differences were determined by one-way ANOVA with Dunnett’s post hoc comparison (*, p<0.05; **, p<0.01 vs. CACL1-Hygro). (C) TSC1 inhibits cell proliferation. Representative phase-contrast micrographs (40× magnification) of established cell lines from 1 day to 4 days after seeding are shown. The displayed data were obtained from the first set; similar results were obtained from the second set (data not shown). (D, E) Cell proliferation was measured by cell counting (D) and an XTT assay (E) and expressed as a fold increase compared to initial absorbance that was measured 4 hours after seeding. Points, mean (n  = 3); bars, SD. Significant differences were determined by Student’s t-test (*, p<0.05; **, p<0.01 vs. CACL1-Hygro, ***, p<0.01 vs. CACL1-YFP).

The function of TSC1 was analyzed in CACL1-TSC1 cell lines that stably express TSC1; these cell lines were produced by transfection of the plasmid pIRES-Hygro-rTSC1 into *Tsc1*-deficient CACL1-111 cells. The mTOR signaling pathway was investigated in the established cell lines by examining the phosphorylation status of S6K and Akt (Fig. 1A, B). As previously reported, TSC1-expressing cells showed a decrease in the levels of phospho-S6K (pS6K) [4], [5], [6] and an increase in those of phospho-Akt (pAkt) [7], [8]. The amount of TSC2 protein increased when TSC1 was expressed, a finding consistent with the TSC1-dependent stabilization of TSC2 reported previously [30], [31]. These results indicate that the mTOR signaling pathway activated by *Tsc1*-deficiency in CACL1-111 cells was suppressed by restoration of TSC1 expression [32]. Restoration of TSC2 suppresses cell proliferation in *Tsc2*-deficient tumor cells [33], [34]. Moreover, TSC1 also has anti-proliferative effects on human cervical cancer (HeLa) cells [35]. Here, proliferation of TSC1-expressing cell lines was examined (Fig. 1C). Control CACL1-Hygro and -YFP (yellow fluorescent protein) cells reached 90% confluence 3 days after seeding, whereas TSC1-expressing cells were only about 60% confluent (Fig. 1C). Inhibition of cell proliferation was significantly greater in CACL1-TSC1 cell lines than in CACL1-Hygro and -YFP cells (Fig. 1D, S1). Similar results were observed in the sodium 3′-[1-(phenylaminocarbonyl)-3, 4-tetrazolium]-bis (4-methoxy-6-nitro) benzene sulfonic acid hydrate (XTT) assay (Fig. 1C, S1B).

### Rac1 is Downregulated by TSC1 without Apparent Changes in Rho Activity

**Figure 2:**
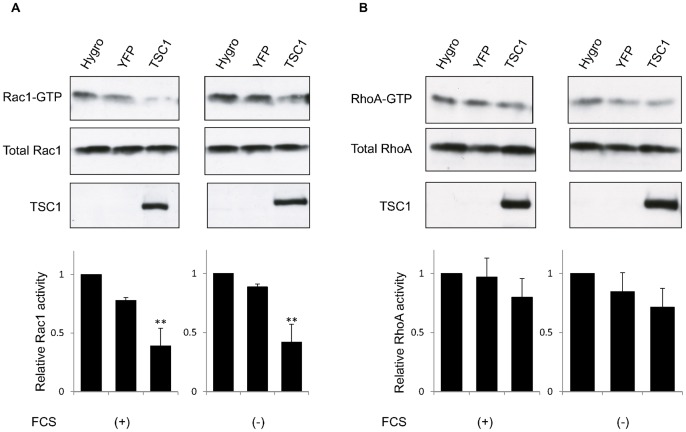
Rac1 is downregulated by TSC1 without apparent changes in Rho activity. (A, B) Cells were subjected to the Rac1 (A) or RhoA (B) activity assay after culturing in normal (FCS+) or serum starvation conditions (FCS−). Western blot analysis was performed to detect Rac1 bound to PAK-1 PBD or rhotekin-PBD beads (top images) and in whole cell lysates (middle images). TSC1 expression was confirmed by immunoblotting of whole cell lysates (bottom images). Representative blots are shown. Quantifications of Rac1 (A) or RhoA (B) activity were performed using Image J (bottom). Levels of active Rac1 (A) or RhoA (B) were normalized to total Rac1 or RhoA, respectively, and expressed as a fold activation relative to CACL1-Hygro cells. Data are shown as means ± SE of three independent experiments. Significant differences were determined by one-way ANOVA with Dunnett’s post hoc comparison (**, p<0.01 vs. CACL1-Hygro).

The effect of TSC1 on Rac1 and Rho GTPases has been studied [20], [29]. Lamb et al. found that overexpression of TSC1 increased the level of RhoA-GTP [20], while Goncharova et al. showed that down-regulation of TSC1 with siRNA stimulated Rac1 activity in TSC2 deficient cells [29]. To test whether expression of TSC1 changes Rho GTPases activity, the activation status of Rac1 and RhoA were examined in the various cell lines used here (Fig. 2, S2). When TSC1 was expressed in *Tsc1*-deficient cells, the amount of active Rac1 was reduced (Fig. 2A, S2A), in agreement with the findings of previous studies [29]. However, the present results differed from previous reports in finding little or no change in RhoA activity in TSC1 expressing cells (Fig. 2B, S1D) [20], [29]. A possible explanation for this apparent discrepancy in the outcome of the experiments may be the use of different cells types. Collectively, the data show that TSC1 inhibits Rac1 activity, but does not substantially affect RhoA activity in renal tumor cells derived from mice.

### TSC1 Inhibits Cell Migration and Formation of Filopodia

**Figure 3:**
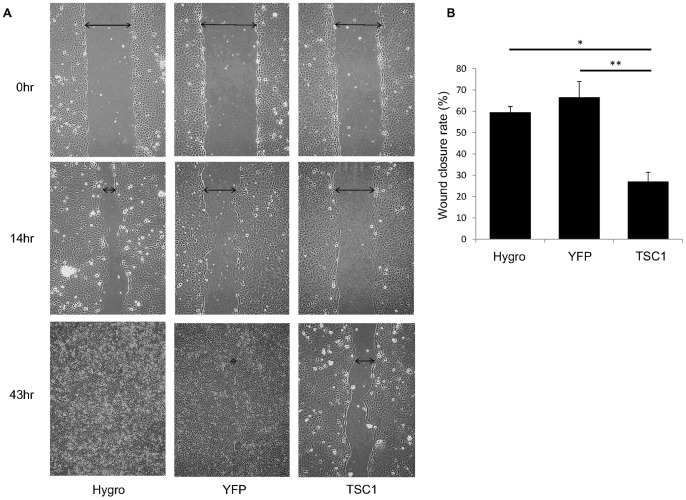
TSC1 inhibits cell migration. (A) TSC1-deficient and TSC1-expressing cells were seeded and grown to 100% confluence. The cell monolayer was scratched with a sterile pipette tip. The wounded cultures were photographed after 14 and 43 hours. Arrows indicate the gap width. Representative images are shown. (B) The rate of wound healing (%) after 14 hours was calculated as described in the Materials and Methods. Data are shown as means ± SE of three independent experiments. Significant differences were determined by one-way ANOVA with Scheffe’s post hoc comparison (*, p<0.05; **, p<0.01).

The effect of TSC1 on cell migration was examined by a scratch wound assay. When cells were grown to confluence and scratched (Fig. 3, S3), migration of TSC1-expressing cells was significantly reduced compared with TSC1-deficient cells.

**Figure 4:**
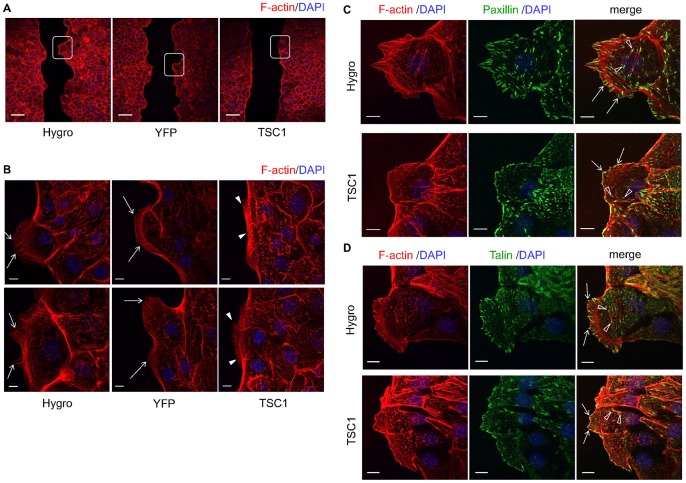
Formation of lamellipodia and filopodia decreases at the leading edge in TSC1-expressing cells. (A) Low-magnification confocal images of phalloidin stained cells. Boxed areas indicate the leading edge. Scale bars: 100 µm. (B) High-magnification images of the leading edge. Two representative images are shown. In each panel, left side is the scratch edge. Arrows indicate the actin fibers in the filopodia of CACL1-Hygro cells and -YFP cells. Arrowheads show actin fibers in the filopodia of CACL1–TSC1 cells. Scale bars: 10 µm. (C, D) TSC1 inhibited focal adhesion formation without changing its structure. Scratched cells were stained for F-actin and components of focal adhesion. The right panels are merged images of F-actin and paxillin (C) or talin (D). Focal complexes appear as small dot-like structures (arrows). Open arrowheads indicate the focal adhesions. Paxillin (C) and talin (D) were observed to associate with both ends of actin stress fibers. Scale bars: 10 µm.

Cell migration is driven by polymerization of a network of cytoskeletal actin filaments and attachment to the extracellular matrix through actin-rich adhesive structures [23]. Here, the front of cell migration (leading edge) was examined by staining F-actin using rhodamine conjugated phalloidin (Fig. 4). At the leading edge of migrating cells, actin assembly drives the extension of flat membrane protrusions called lamellipodia and filopodia. In TSC1-expressing cells, formation of lamellipodia and filopodia appeared to be decreased compared to TSC1-deficient cells (Fig. 4A, B, S4A, B). Linear actin filaments in the filopodia were perpendicular to the margins of the leading edge in CACL1-Hygro and -YFP cells (Fig. 4B, S4B). By contrast, actin filaments in CACL1-TSC1 cells appeared to be shorter and were aligned obliquely to the edge (Fig. 4B, S4B).

An immunocytochemical analysis was then performed to investigate the focal adhesion remodeling at the leading edge that is essential for cell migration (Fig. 4C, D, S4C, D). Paxillin and talin were colocalized with F-actin and appeared as small dot-like structures at the cell periphery of the leading edge (Fig. 4C, D, S4C, D). These structures seemed to be focal complexes. Separate to the focal complexes, paxillin and talin formed structures known as focal adhesions that associated with both ends of the actin stress fibers (Fig. 4C, D). The number of focal adhesions seemed to be smaller in CACL1-TSC1 cells compared with CACL1-Hygro cells, although there was little overall difference in their structures among the cell lines. These results demonstrate that cell migration is inhibited by TSC1 probably as a consequence of the reduction of filopodia and focal adhesions.

### TSC1 Contributes to Induction of the Apical Actin Fibers

**Figure 5:**
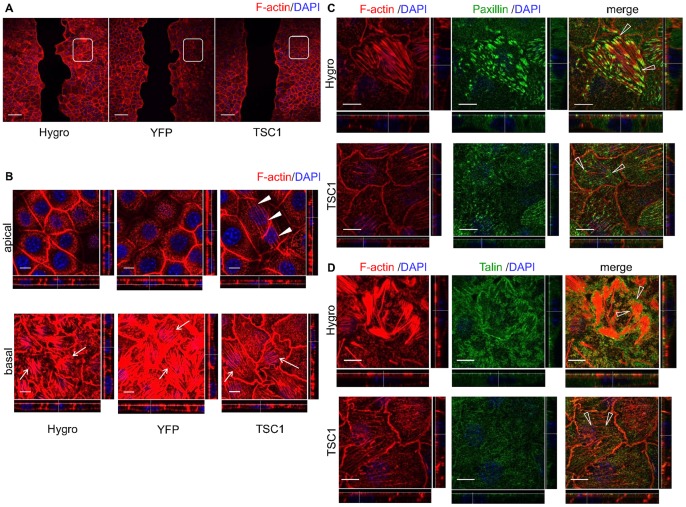
TSC1 reduces basal actin fibers and induces apical actin fibers in the confluent monolayer. (A) Low-magnification confocal images of phalloidin stained cells. Boxed areas show the confluent monolayer. Scale bars: 100 µm. (B) TSC1 reduced basal actin fibers and induced apical actin fibers. Images show representative X–Y sections from scans at 0.5 µm steps from the basal (close to the substrate, lower panels) to the apical side (upper panels) of the cell. X–Z (top to bottom) and Y–Z projections (left to right) are shown at the bottom and right side of each panel, respectively. Dotted line indicates the level of X–Y images shown. Arrows denote actin stress fibers in the basal side of cells. Arrowheads indicate the actin fiber network in the apical side of CACL1-TSC1-11cells. Scale bars: 10 µm. (C, D) TSC1 inhibited formation of focal adhesions in the confluent stage. Cells in confluent monolayer were stained for F-actin and paxillin (C) or talin (D). Open arrowheads show focal adhesions connected to stress fibers. Scale bars: 10 µm.

The actin cytoskeleton was examined in more detail in cells distant to the scratch in the confluent monolayer (Fig. 5, S4E). Actin stress fibers were thick and irregular in the basolateral side of CACL1-Hygro and -YFP cells (Fig. 5B, S4E). In contrast, CACL1-TSC1 cells contained thin, but well-organized stress fibers (Fig. 5B, S4E). These basal actin fibers were connected to focal adhesions at both ends in CACL1-Hygro and TSC1 cells (Fig. 5C, D, S4F, G). Focal adhesions were more abundant in TSC1-deficient cells than in TSC1-expressing cells, an observation similar to that described above for the leading edge. These results suggest that formation of focal adhesions and stress fibers is inhibited by TSC1 in the confluent stage.

Intriguingly, TSC1-expressing cells showed aligned actin fibers that traversed the apical region (Fig. 5B, S4E); these structures were never seen in TSC1-deficient cells. The aligned actin fibers originated from cortical actin and were localized at cell-cell junctions. The structure composed by these actin fibers is termed here the apical actin network, because the fibers traverse the cells lengthwise and appear to be intercellularly connected (Fig. 5B, S4E). Apical actin networks could be seen in nearly all TSC1-expressing cells in the confluent monolayer region, but not in cells at the leading edge, suggesting that they may have a role in the formation of the epithelial structure.

### Apical Actin Network Associates with TJs

**Figure 6:**
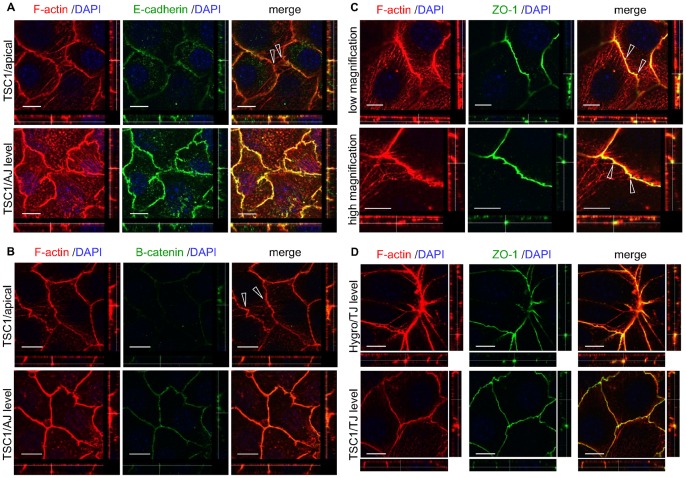
The apical actin network associates with TJs in CACL1-TSC1 cells. (A, B) Apical actin fibers originate more apically of the adherens junction (AJ). The images were analyzed as described in Figure 5. Cells in confluent monolayer were stained for F-actin and E-cadherin (A) or β-catenin (B). Open arrowheads show the apical actin network. Scale bars: 10 µm. (C) Apical actin network associated with TJs in CACL1-TSC1 cells. CACL1-TSC1-11 cells in confluent monolayer were stained for F-actin and the TJ component ZO-1. The images were analyzed as described in Figure 5. Lower panels show higher magnification photographs. Open arrowheads show the junction of the apical actin network and ZO-1. The shape of the intercellular junctional perimeter was changed as if pulled by apical actin fibers. (D) There was no apparent change in TJ formation in the TSC1- deficient cells compared to the TSC1- expressing cells. ZO-1 in CACL1-Hygro cells was analyzed using the same method as for CACL1-TSC1 cells. Similar observations were obtained from the second clone set (data not shown). Scale bars: 10 µm.

AJs and TJs are important for intercellular contacts, and both are thought to be associated with the actin cytoskeleton [36]. The relationship between the apical actin network and these intercellular junctions was investigated by immunostaining components of AJs and TJs in CACL1-TSC1 cells. The apical actin fibers did not colocalize with the components of the AJ components such as E-cadherin and β-catenin, but seemed to originate more apically of these structures (Fig. 6A, B). However, the apical actin fibers were often associated with ZO-1, a component of TJs (Fig. 6C). The shape of the intercellular junctional perimeter was suggestive of tension exerted by these apical actin fibers. Taken together, these results indicate that the apical actin fiber network originated from TJs in TSC1-expressing cells. Additionally, ZO-1 was not increased in CACL1-TSC1 cells compared to CACL1-Hygro cells (Fig. 6D), suggesting that the apical actin network did not result from an increase in TJs.

### TSC1 Promotes the Formation of Apical Actin Network through Spatial Regulation of Rho Family of Small GTPases

**Figure 7:**
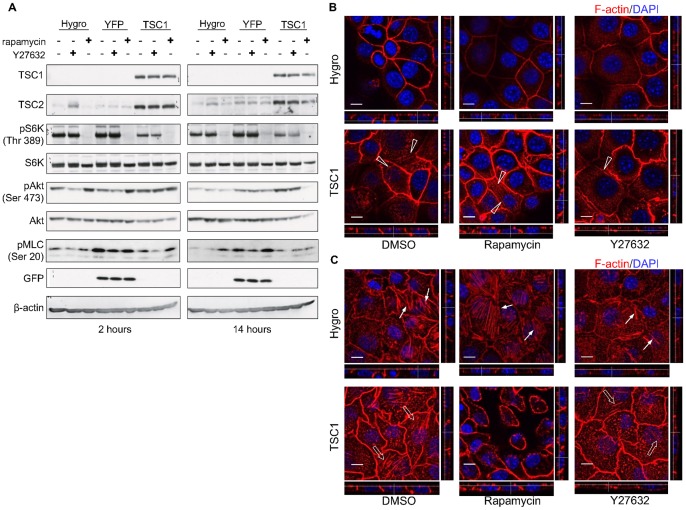
TSC1 induces the formation of apical actin network in a RhoA-dependent manner independently of mTOR. (A) mTORC1 and mTORC2 were inhibited by rapamycin treatment but were not changed by ROCK inhibitor treatment. Cells were harvested and lysed 2 (left panels) or 14 hours (right panels) after inhibitor addition. Total cell lysates were analyzed by immunoblotting with the indicated antibodies. (B) Apical side of cells. Apical actin network remained unchanged by inhibition of mTORC1/2 and was decreased after inhibition of ROCK. Open arrowheads indicate the actin fiber network in CACL1-TSC1 cells. (C) Basal side of cells. Basal actin stress fibers were decreased after inhibition of ROCK regardless of TSC1. Arrows and open arrows show the basal actin stress fibers in CACL1-Hygro and -TSC1 cells, respectively. Confluent cells were treated with rapamycin or Y27632 for 14 hours and stained for F-actin. Images were analyzed as described in Figure 5. In (B) and (C), scale bars: 10 µm.

In order to investigate possible mechanisms for the formation of the apical actin network, the cell lines were treated with rapamycin or Y27632, which inhibit mTOR and Rho-kinases (ROCKs), respectively. With respect to the mTOR signaling pathway, phosphorylation of S6K was markedly decreased by rapamycin treatment in all cell lines (Fig. 7A). The level of pAkt was significantly increased by a 2 hour treatment of TSC1-deficient cells with rapamycin, possibly because of the inhibition of a negative feedback loop on insulin receptor substrate-1 (IRS1) [7], [8]. After a 14 hour treatment, however, pAkt levels decreased, indicating that mTORC2 was also inhibited by the longer rapamycin treatment in TSC1-expressing cells, as has been reported elsewhere [37], [38]. Treatment with Y27632 did not induce any apparent change in the phosphorylation levels of S6K or Akt, whereas it reduced phosphorylation of myosin light chain (MLC).

The cells were then immunocytochemically stained to visualize the effects of the inhibitors on the apical actin network (Fig. 7B, C). In this experiment, the inhibitor was added to the culture medium immediately after scratching, and the cells were fixed after 14 hours. The number of apical actin fibers was markedly reduced in Y-27632-treated CACL1-TSC1 cells (Fig. 7B). Furthermore, the number of basal actin fibers also decreased after Y-27632 treatment in both TSC1-deficient (Fig. 7C) and TSC1-expressing cells (Fig. 7C). By contrast, rapamycin had no effect on the formation of the apical actin network in TSC1-deficient cells or in TSC1-expressing cells (Fig. 7B). These results indicate that TSC1 contributes to the formation of the apical actin network in epithelial cells in a ROCK-dependent manner independently of mTOR.

**Figure 8:**
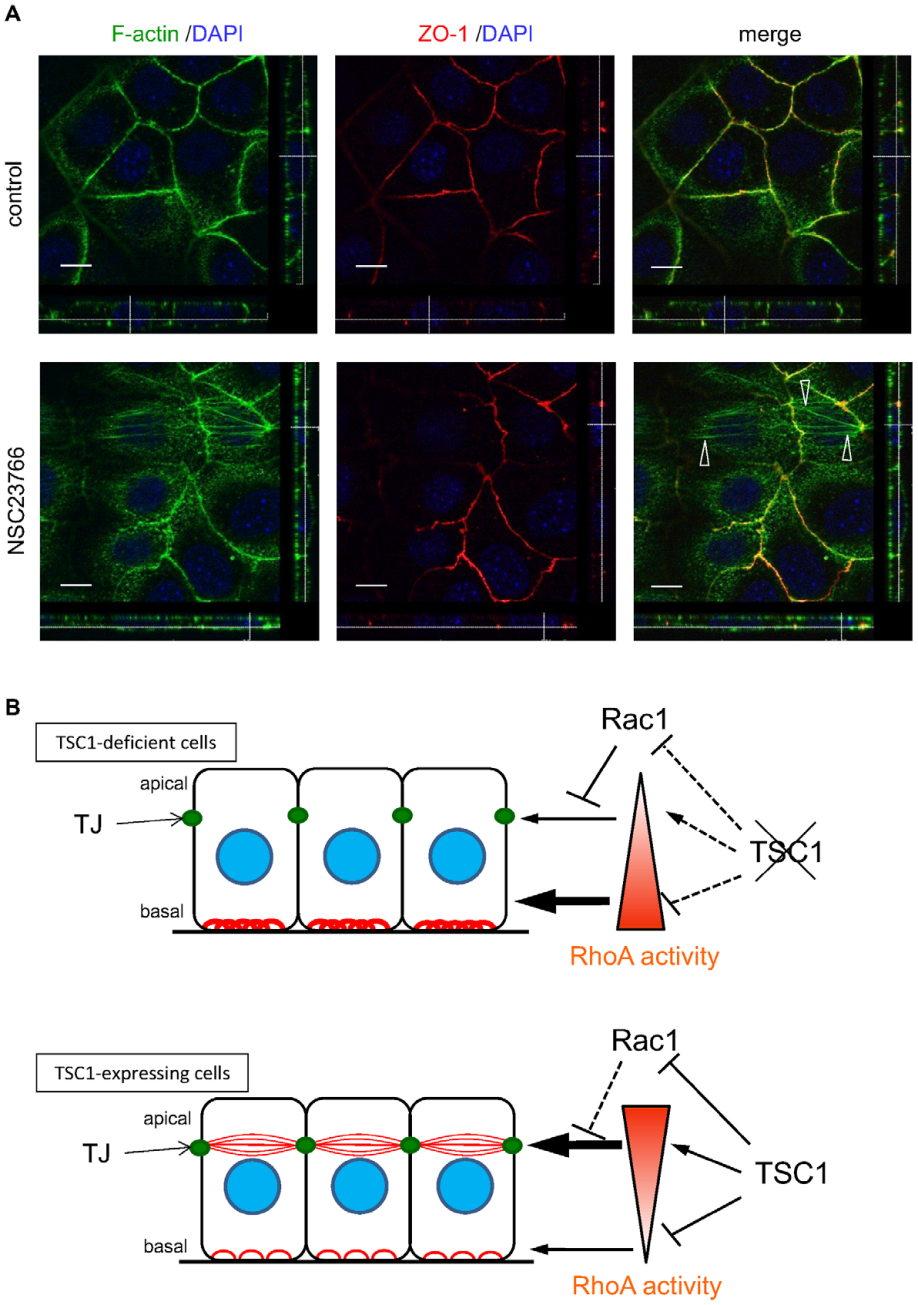
Inhibition of Rac1 led to the formation of apical actin network. (A) NSC23766, a Rac1 inhibitor, induced TJ-associated apical actin fibers in TSC1-deficient cells. TSC1-deficient cells (CACL1-Hygro), plated 24 hours prior to treatment, were cultured in the absence (upper images) or presence of NSC23766 (lower images) for 3 days. Open arrowheads indicate apical actin fibers at TJ level. Scale bars: 10 µm. Similar results were obtained from the second clone set (data not shown). (B) Model of TSC1 function during actin cytoskeletal change in CACL1-111 cells. Red lines show actin fibers. Stress fibers in the basolateral side decreased and the apical actin network appeared in TSC1-expressing cells. RhoA activity (depicted by the triangle) is suspected to be high in the apical side and low in the basolateral side of TSC1-expressing cells. Thus, spatial regulation of RhoA activity may be regulated by TSC1. Rac1 may inhibit RhoA [29], [52] and downregulation of Rac1 by TSC1 may also contribute to the apical actin network.

Finally, to determine whether suppression of Rac1 activity by TSC1 expression played a role in the development of the apical actin network, TSC1-deficient cells were treated with the Rac1 inhibitor NSC23766 (Fig. 8A). Actin fibers that traversed the apical region of TSC1-deficient cells appeared after inhibition of Rac1, indicating that repression of Rac1 activity was essential for development of the apical actin network.

## Discussion

The *Tsc1*-deficient renal tumor cell line used here to study the function of TSC1 was originally established from a *Tsc1* knockout mouse [4], [39]. The analysis of the mTOR signaling pathway showed that pS6K levels were decreased in TSC1-expressing cells, as has been reported previously [4], [5], [6]. Expression of TSC1 increased the amount of TSC2 protein in the cells, an effect that is consistent with the previously reported TSC1-dependent stabilization of TSC2 [30], [31]. These results indicate that excessive activation of mTORC1 signaling pathway due to *Tsc1*-deficiency in mouse renal tumor cells was abrogated by restoration of TSC1. pAkt levels were also observed to be increased by TSC1 expression and by a 2 hour treatment with rapamycin in TSC1-deficient cells. Aberrant activation of mTORC1 downregulates IRS1 through a negative feedback loop, thereby inhibiting phosphorylation of Akt [7], [8]. The results here suggest that this negative feedback loop was enhanced during renal tumorigenesis in *Tsc1* knockout mice, as has been found in *Tsc2* knockout mice [7], [8].

Restoration of TSC1 was found here to inhibit cell proliferation. This finding is consistent with previous studies which showed that TSC1 and TSC2 control cell proliferation. Initially, several studies reported that TSC2 inhibits cell proliferation in TSC2-deficient rat renal carcinoma cell lines [33], [34]. Of note, the C-terminal GAP domain of TSC2 is sufficient for the suppression of proliferation and tumorigenesis [33]. Moreover, TSC1 has been reported to have anti-proliferative effects in HeLa cells [35] and in *Drosophila* [40]. The present results are in agreement with these previous studies, and indicate that TSC1 also regulates cell proliferation in this mouse renal tumor cell line.

In this study, migration of TSC1-expressing cells was found to be significantly reduced compared with TSC1-deficient cells. Several previous studies have indicated that TSC1 and TSC2 may play a role in regulating cell migration. Overexpression of TSC2 in epithelial cells such as MDCK leads to a decrease in cell migration [41]. Conversely, a later study showed that TSC2 potentiates cell migration in rat embryonic fibroblasts [42]. Thus there are inconsistencies among the published studies; these inconsistencies may be a consequence of differences in the experimental methods or cell types. For TSC1, it was reported that the nestin-positive cell-specific loss of *Tsc1* resulted in aberrant cell migration in the brains of *Tsc1* conditional knockout mice [43]. To date, however, there have been no reports that TSC1 functions in cell migration *in vitro*, particularly in epithelial cells. To our knowledge, this is the first report of a TSC1 function in cell migration in mammalian epithelial cells.

The driving force for cell migration is provided by the reorganization of the actin cytoskeleton, a dynamic and complex process orchestrated by activation of Rho family small GTPases. Rac1 is essential for the protrusive activity of the cell edge and Rho is required for stress fiber formation and maintenance of cell-substrate adhesion (i.e., focal complexes and focal adhesions) [44]. Here, we found that formation of lamellipodia and filopodia decreased at the leading edge in TSC1-expressing cells, and that TSC1 inhibited Rac1 activity. These data indicate that TSC1 inactivates Rac1, thereby reducing cell motility by suppression of the formation of protrusions at the leading edge.

In this study, the re-expression of TSC1 in TSC1-deficient mouse renal tumor cell lines markedly inhibited the assembly of stress fibers and focal adhesions without loss of the architectural relationship between them. These results are inconsistent with previous studies showing that TSC1 promotes their assembly [20], [29]. The reasons for the discrepancy between the previous reports and the present study are not clear, but one possible explanation is the difference in the cell types used in the studies. Lamb et al. used human umbilical vein endothelial cells (HUVEC) and mouse fibroblasts [20], whereas we used mouse renal tumor cell lines. The discrepancy may therefore be due to the differences between endothelial cells and epithelial cells, or non-polarized cells and polarized cells. Goncharova et al. analyzed the function of TSC1 using TSC2-deficient cells [29], whereas the present study was conducted in the presence of TSC2. This raises the possibility that the TSC2 status might be responsible for the inconsistent results.

In the confluent monolayer, we identified aligned apical actin fibers that traversed the cell body in the apical region of TSC1-expressing cells. This “apical actin network” seemed to connect with cortical actin at the level of the TJs (Fig. 8A). The shape of the TJ perimeter in relation to the apical actin network was suggestive of tension exerted by the fibers. Apical cell-cell junction-associated actin fibers similar to this apical actin network have been described in previous reports, although their descriptions are not entirely consistent with those here [45], [46], [47], [48], [49]. The tension generated by this type of actin fiber is important in determining the relationship between the AJ and actin fibers [45]. It was previously reported that parallel stress-fiber-like bundles co-localized with the ZO-1 in non-polarized cells, but were less developed in polarized cells. The cells used here were polarized, irrespective of the presence or absence of an apical actin network. The mechanism for forming the apical actin network in polarized cells may differ from that in non-polarized cells. Most previous studies reported that this type of apical actin fiber was associated with AJ components, such as cadherin or catenins [45], [46], [47], [48], [49]; here, however, we found that the apical actin network clearly colocalized with ZO-1. It has also been reported that radially organized actin fibers appeared when cell-cell junction formation was inhibited [46]. In contrast to that report, there was no apparent change in cell-cell junction formation in the TSC1-expressing cells compared to the TSC1-deficient cells. This observation suggests that the formation of the apical actin network is not directly related to the assembly and disassembly of cell-cell junctional complexes.

In neuroepithelial cells, apical constrictions provoked by apical actin fibers are important for neural tube closure [47]. In keratinocytes, radial actin cables in the apical zone, similar in appearance to an actin fiber network, transiently appear in the early steps of stratification [46]. Additionally, an actin cytoskeletal structure similar to the apical actin network has been reported in endothelial cells [49]. From the observation here that the fibers were arranged as if they were intercellularly connected, we speculate that the functions of the apical actin network might be coordinated between different cells.

The cell lines were treated with inhibitors in an attempt to identify characteristics of the mechanisms that underlie the formation of the apical actin network. The apical actin network was not induced by rapamycin treatment of TSC1-deficient cells, suggesting that it is formed in an mTORC1-independent manner. However, the apical actin network was not reduced by long-term rapamycin treatment in TSC1-expressing cells. With regard to Akt phosphorylation, it appears that mTORC2 is upregulated by TSC1 expression [50], [51], and inhibited by long-term rapamycin treatment. This suggests that the apical actin network formation does not depend on mTORC2. The phosphorylation of Akt, once induced by short-term rapamycin exposure, is canceled after longer treatment, indicating that rapamycin inhibits not only mTORC1 but also mTORC2 [37], [38]. Hence, the apical actin network seen in TSC1-expressing cells is formed independently both of mTORC1 and mTORC2. After Y27632 treatment, however, the apical actin network was markedly decreased. This suggests that apical actin formation was mediated by ROCK, which is one of the major targets of RhoA. Likewise, actin stress fibers in the basolateral side of cells were also decreased by Y27632 treatment in both TSC1-deficient cells and TSC1-expressing cells. The decrease in TSC1-deficient cells may be similar to that caused by TSC1 expression. Overall, RhoA activity is suspected to be high in the apical side and low in the basolateral side of TSC1-expressing cells. Although the amount of active RhoA in whole cells was not found to be increased, it is plausible that spatial regulation of active RhoA localization is regulated by TSC1. This may explain why this type of apical actin was attenuated after inhibition of RhoA. In other words, our results suggest that TSC1 enhances RhoA activity and actin fiber formation in the apical region of cells. We hypothesize, on the basis of a previous report [20] and our present observations, that the enhancement of actin fiber formation by TSC1 might occur in both the basolateral and apical sides of cells depending on the specific cell-type context. The present results show distinctive regulation of cell polarity in mouse renal tumor cells.

The downregulation of Rac1 in TSC1-expressing cells may also contribute to the apical actin network. In our study, treatment of TSC1-deficient cells with Rac1 inhibitor led to development of apical actin fibers, which were similar to those seen in TSC1-expressing cells. This suggests that Rac1 inhibition is essential for the formation of apical actin network. Collectively, TSC1 promotes the apical actin network via inhibition of Rac1 activity. In this context, a recent study has shown that RhoA promotes apical constriction through the production of phospho-myosin regulatory light chains, whereas Rac1 suppresses this function of RhoA [52]. Taken together, we propose a model for the cell polarity-dependent regulation of actin cytoskeleton by TSC1 (Fig. 8B). It is conceivable that the apical actin network in TSC1-expressing cells is induced by the predominance of RhoA over Rac1 activity.

In conclusion, this work has demonstrated that TSC1 regulates the actin cytoskeleton via a novel pathway, thereby playing a role in cell migration and polarity-associated morphological regulation. The cell lines described in this report will be instrumental in elucidating molecular mechanisms underlying the function of TSC1 in the regulation of actin cytoskeleton in the renal tumors.

## Materials and Methods

### Plasmid Construction

A DNA fragment containing the coding region of N-terminally HA-tagged rat hamartin was excised from the expression plasmid pCAG-HA-rTsc1 by digestion with *Xba*I and *Bgl*II [53], [54]. The fragment was inserted into the *Nhe*I-*Bam*HI site of a pIRES-hyg3 vector (Clontech, Palo Alto, CA), generating pHArTSC1-IRES-hyg. The expression plasmid for YFP was constructed by digesting the pEYFP-N1 vector (Clontech) with *Not*I and using T4 DNA polymerase for end filling. The coding fragment for EYFP was then excised by *Nhe*I digestion and subcloned into the *Nhe*I-*Eco*RV site of the pIRES-hyg3 vector, generating pEYFP-IRES-hyg.

### Cell Culture and Generation of Stable Cell Lines Expressing Tsc1

CACL1-111 cells, a *Tsc1*-deficient cell line derived from a renal tumor of *Tsc1* knockout mice [4], [39], were maintained in RPMI 1640 medium (Sigma-Aldrich, St. Louis, MO) supplemented with 10% fetal calf serum (FCS). To establish hygromycin B-resistant stable cell lines, CACL1-Hygro, CACL1-YFP and CACL1-TSC1, pIRES-Hygro, pIRES-YFP or pIRES-Hygro- rTSC1 were transfected into CACL1-111 cells using electroporation. Briefly, 20 µg of plasmid DNA was mixed with 4×10^6^ CACL1-111 cells in 0.4 ml of medium in a 4 mm gap cuvette and immediately pulsed (200 V, 70 ms pulse length, one pulse) using a BTX ECM 830 square wave electroporation system (Genetronics, San Diego, CA, USA). Cells were selected in RPMI 1640 medium containing 10% FCS and 300 µg/ml hygromycin B. Finally, cell lines were single-cell cloned by limiting dilution. To obtain independent clones, genomic Southern blot analysis was performed.

In some experiments, cells were treated with 20 nM rapamycin (Sigma-Aldrich), 10 µM Y27632 (Wako Pure Chemical Industries, Osaka, Japan) or 100 µM NSC23766 (Calbiochem, San Diego, CA, USA).

### Western Blotting

Cells were harvested and lysed in standard SDS-PAGE sample buffer (50 mM Tris-HCl, pH6.8, 2% SDS, 10% glycerol). Proteins were separated by SDS polyacrylamide gel electrophoresis (SDS-PAGE) and transferred onto polyvinylidene fluoride (PVDF) membrane (Millipore, Billerica, MA, USA), blocked with 1% skim milk in Tris-buffered saline containing 0.05% Tween 20, and probed with the appropriate antibodies, using the Envision system (Dako, Denmark) [55]. Antibody signals were developed with the ECL prime Western blotting detection reagents (GE Healthcare), and the membrane was exposed to Amersham Hyperfilm ECL films (GE Healthcare) which were then scanned using CEPROS SV (Fujifilm Co.). The following primary mouse monoclonal (mAb), rabbit monoclonal (rmAb) and rabbit polyclonal (pAb) antibodies were used: anti-TSC1 pAb (1∶300 dilution) [30], anti-TSC2 pAb (C20; 1∶500, Santa Cruz Biotechnology, Santa Cruz, CA, USA), anti-p S6K (Thr389) rmAb (108D; 1∶1000, Cell Signaling Technology, Beverly, MA), anti- S6K pAb (C-18; 1∶500, Santa Cruz Biotechnology), anti-pAkt (Ser473) rmAb (D9E; 1∶1000, Cell Signaling Technology), anti-Akt pAb (1∶1000, Cell Signaling Technology), anti-Rac1 pAb (C-11; 1∶1000, Santa Cruz Biotechnology), anti-RhoA mAb (1∶500, Cytoskeleton, Denver, CO), anti-GFP pAb (1∶100, Clontech Laboratories, Palo Alto, CA), anti-p MLC (Ser20) pAb (ab24801; 1∶5000, Abcam, Cambridge, UK) and anti-ß-actin mAb (1∶5000) (Sigma-Aldrich).

### Cell Proliferation Assay

Cell proliferation was determined by cell counting and XTT assay. To determine cell proliferation by counting cell numbers, the cells were seeded on a 6 cm dish at a density of 1×10^5^ cells/dish. Cells were collected with Accumax (Innovative Cell Technologies Technologies Inc., San Diego, CA), and counted every 24 hours. To monitor cell proliferation by XTT assay, the Cell Proliferation Kit II XTT (Roche Diagnostics) was used in accordance with the manufacturer’s protocol. Briefly, cells were seeded in 96-well plates at a density of 2000 cells/well in a volume of 100 µl. A mixture of XTT (sodium 3′-[1-(phenylaminocarbonyl)-3, 4-tetrazolium]-bis (4-methoxy-6-nitro) benzene sulfonic acid hydrate) and PMS (N-methyl dibenzopyrazine methylsulfate) was added, and the cells were incubated for 2 hours. Absorbance at 492 nm (reference wavelength 690 nm) was measured using a microplate reader (Bio-Rad Laboratories). Proliferation was analyzed daily from day 0 to day 5 after seeding in triplicate for each time point. The proliferation status of the cells, seeded on 6 cm dishes at a density of 2×10^5^ cells/dish, was recorded by phase-contrast micrography every day using an Olympus digital camera (Olympus, Tokyo, Japan).

### Rac1 and RhoA Activity Assay

The level of active GTP-bound Rac1 or RhoA was determined using a Rac1 or Rho Activation Assay Biochem kit (Cytoskeleton) according to the manufacturer’s instructions. TSC1-deficient or -expressing cells were seeded on a 10 cm dish at a density of 4 or 10×10^5^ cells/dish, respectively. Forty-eight hours after seeding, culture dishes were cooled on ice, washed with cold phosphate buffered saline (PBS) and lysed with ice-cold cell lysis buffer. Cell lysates were cleared by centrifugation, and active Rac1 or RhoA was precipitated with PAK-PBD or rhotekin-PBD beads, respectively. The precipitated proteins were solubilized in SDS sample buffer and analyzed by Western blotting. Serum starvation conditions were obtained by replacing the growth medium with serum free medium 24 hours after seeding, and the assay was performed after starvation for 24 hours.

### Scratch Wound Assay

Cell migration was examined using the scratch wound assay with cells grown to confluence in 35 mm dishes. The monolayers were scratched using a sterile 200 µl sterile plastic pipette tip, and were then washed with growth medium. The wound was photographed immediately and at 14 and 40 or 43 hours later, and measured using Image J. The percentage of wound healed was then calculated using the following formula: 100 − (final area/initial area ×100%).

### Immunofluorescence and Confocal Microscopy

For confocal microscopy, cells were plated in a 35 mm glass bottom dish treated with Advanced TC™ (Greiner, Bio One GmbH, Frickenhausen, Germany) and scratched as described above. Fourteen hours after scratching, cells were fixed and permeabilized with 4% paraformaldehyde (Wako Pure Chemical Industries, Ltd.) and 0.25% Triton X-100 (Wako Pure Chemical Industries) in PBS for 30 min at 4°C, and then given 3×5 min washes with PBS/0.1% bovine serum albumin. Cells were incubated with a primary antibody and rhodamine- or BODIPY-labeled phalloidine (Molecular Probes, Eugene, OR), diluted in PBS in 1% bovine serum albumin (Iwai Kagaku Co., Tokyo), for 1 hour at room temperature. The cells were then washed and further incubated with fluorophore-conjugated secondary antibody and 4′, 6-diamidino-2-phenylindole (DAPI) for 1 hour at room temperature. Immunofluorescence images were collected using a Leica TCS SP5 ver2.0 system equipped with a 63×/1.40 NA water-immersion and 100×1.40/NA oil-immersion objective lens (Leica, Heidelberg, Germany). The following primary antibodies were used: anti-E-cadherin mAb (1∶50 dilution for use, BD Biosciences), anti-talin mAb (1∶500, Sigma-Aldrich), anti-paxillin mAb (1∶200, BD Biosciences), anti-β-catenin pAb (1∶1000, Upstate Biotechnology, Lake Placid, NY, USA), and anti-ZO-1 mAb (1∶100, Sanko Junyaku Co., Ltd., Tokyo, Japan). Alexa Fluor (488 or 568)-conjugated goat anti-mouse or goat anti-rabbit secondary antibodies (Molecular Probes) were used at 1∶1000 dilution.

### Statistical Analysis

Statistical analysis was performed by Student’s t test or one-way ANOVA followed by Dunnett’s or Scheffe’s post-hoc test using Ekuseru-Toukei 2010 (Social Survey Research Information Co. Ltd., Tokyo, Japan). P values of less than 0.05 were considered statistically significant.

## Supporting Information

Figure S1 TSC1 inhibits cell proliferation of TSC1-deficient cells in another independent clone set. (A, B) Cell viability and proliferation was measured by cell counting (A) and XTT assay (B) in a second independent clone set. Points, mean (n  = 3); bars, SD. Significant differences were determined by Student’s t-test (*, p<0.05; **, p<0.01 vs. CACL1-Hygro, ***, p<0.01 vs. CACL1-YFP).
(TIF)

Figure S2 Rac1 is downregulated by TSC1, but not Rho activity, in another independent clone set. (A, B) Cells were subjected to the Rac1 (A) or RhoA (B) activity assay after culturing in normal (FCS+) or serum starvation conditions (FCS-). Western blot analysis was performed to detect Rac1 bound to PAK-1 PBD or rhotekin-PBD beads (top images) and in whole cell lysates (bottom images). Representative blots for a single experiment are shown. Quantification of Rac1 (A) or RhoA (B) activity was performed using Image J (bottom). Levels of active Rac1 (A) or RhoA (B) were normalized to total Rac1 or RhoA, respectively, and expressed as fold activation relative to CACL1-Hygro cells. Data are shown as means ± SE of three independent experiments. Significant differences were determined by one-way ANOVA with Dunnett’s post hoc comparison (*, p<0.05 vs. CACL1-Hygro).
(TIF)

Figure S3 TSC1 inhibits cell migration in another independent clone set. (A) The confluent monolayer of TSC1-deficient and TSC1-expressing cells was scratched with a sterile pipette tip. The wounded cultures were photographed after 14 and 40 hours. Arrows indicate the gap width. Representative images are shown. (B) The rate of wound healing (%) after 14 hours was calculated as described in the Materials and Methods. Data are shown as means ± SE of three independent experiments. Significant differences were determined by one-way ANOVA with Scheffe’s post hoc comparison (*, p<0.05).
(TIF)

Figure S4 TSC1 alters actin cytoskeleton not only in the basolateral but also the apical region in cells of another independent clone set. (A) Low-magnification confocal images of phalloidin stained cells. Scale bars: 100 µm. (B) High-magnification images of the leading edge. Representative images are shown. In each panel, left side is the scratch edge. Arrows indicate the actin fibers in the filopodia of CACL1-Hygro cells and -YFP cells. Arrowheads showed actin fibers in the filopodia of CACL1–TSC1 cells. Scale bars: 10 µm. (C, D) Scratched cells were stained for F-actin and components of focal adhesion. Representative merged images of F-actin and paxillin (C) or talin (D). Focal complexes appear as small dot-like structures (arrows). Scale bars: 10 µm. (E) TSC1 reduced basal actin fibers and induced apical actin fibers. Images show representative X-Y sections from scans at 0.5 µm steps from the basal (close to the substrate, lower panels) to the apical side (upper panels) of the cell. X-Z (top to bottom) and Y-Z projections (left to right) are shown at the bottom and right side of each panel, respectively. Dotted line indicates the level of X-Y images shown. Arrows denote actin stress fibers in the basal side of cells. Arrowheads indicate the actin fiber network in the apical side of CACL1-TSC1-11 cells. Scale bars: 10 µm. (F, G) TSC1 inhibited formation of focal adhesions in the confluent stage. Cells in confluent monolayer were stained for F-actin and paxillin (F) or talin (G). Open arrowheads show focal adhesions connected to stress fibers. Scale bars: 10 µm.
(TIF)
