# Supplementary figures and images for: TSC1 Controls Distribution of Actin Fibers through Its Effect on Function of Rho Family of Small GTPases and Regulates Cell Migration and Polarity
Source: PLoS One. 2013 Jan 23;8(1):e54503. doi: 10.1371/journal.pone.0054503 (PMC3552859; doi:10.1371/journal.pone.0054503)

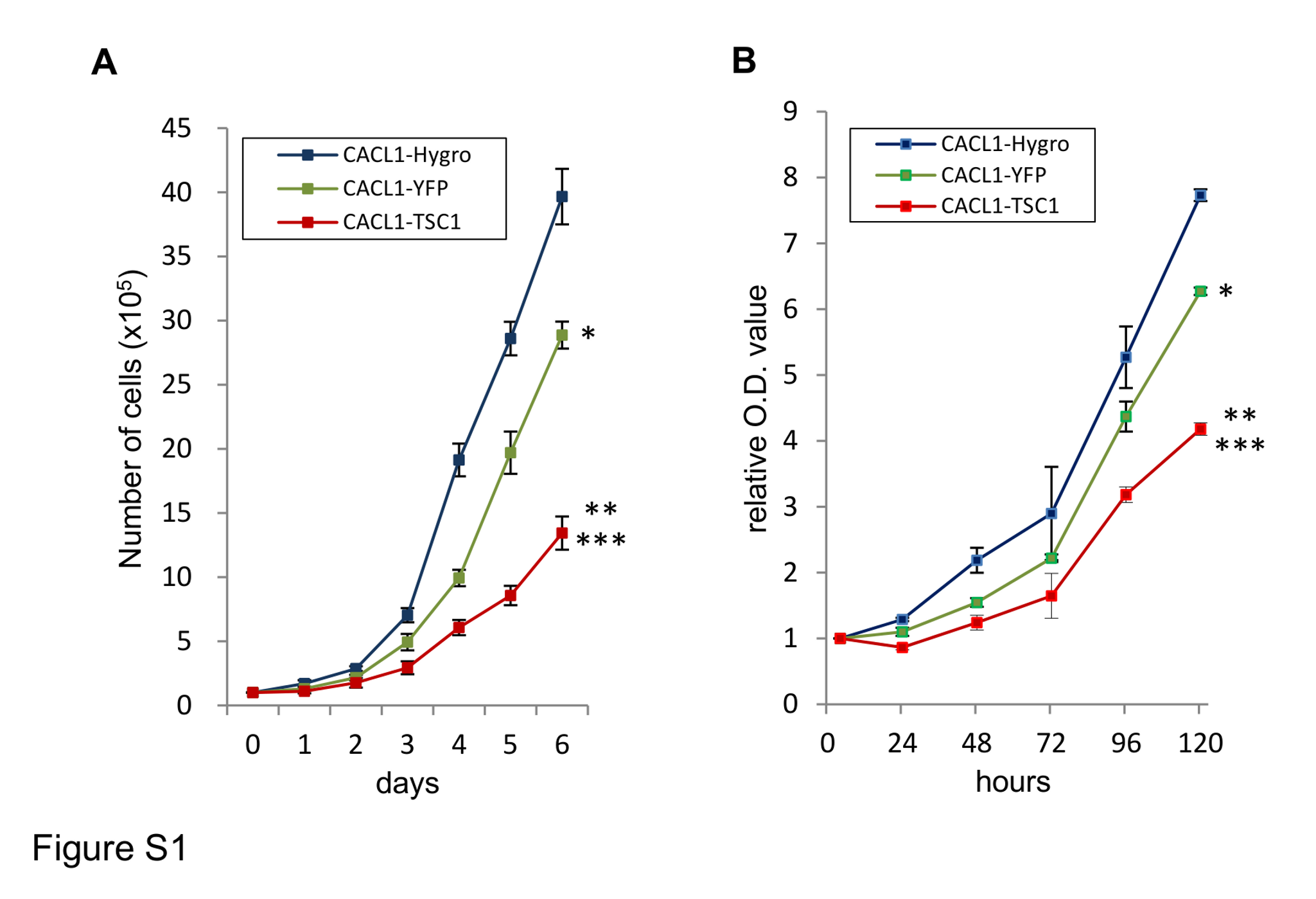

Supplement: Figure S1 — TSC1 inhibits cell proliferation of TSC1-deficient cells in another independent clone set. (A, B) Cell viability and proliferation was measured by cell counting (A) and XTT assay (B) in a second independent clone set. Points, mean (n = 3); bars, SD. Significant differences were determined by Student’s t-test (*, p<0.05; **, p<0.01 vs. CACL1-Hygro, ***, p<0.01 vs. CACL1-YFP). (TIF) [file pone.0054503.s001.tif]

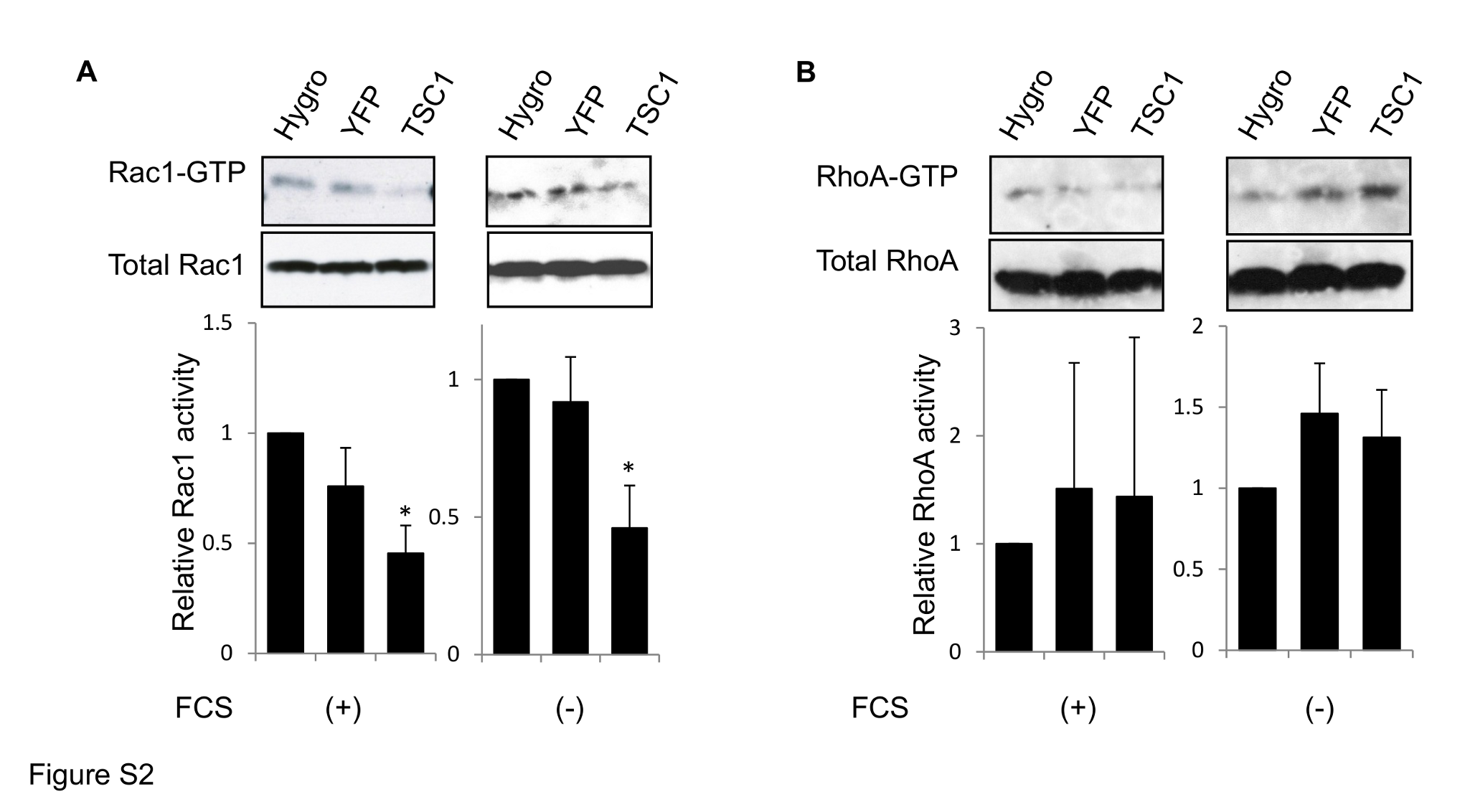

Supplement: Figure S2 — Rac1 is downregulated by TSC1, but not Rho activity, in another independent clone set. (A, B) Cells were subjected to the Rac1 (A) or RhoA (B) activity assay after culturing in normal (FCS+) or serum starvation conditions (FCS-). Western blot analysis was performed to detect Rac1 bound to PAK-1 PBD or rhotekin-PBD beads (top images) and in whole cell lysates (bottom images). Representative blots for a single experiment are shown. Quantification of Rac1 (A) or RhoA (B) activity was performed using Image J (bottom). Levels of active Rac1 (A) or RhoA (B) were normalized to total Rac1 or RhoA, respectively, and expressed as fold activation relative to CACL1-Hygro cells. Data are shown as means ± SE of three independent experiments. Significant differences were determined by one-way ANOVA with Dunnett’s post hoc comparison (*, p<0.05 vs. CACL1-Hygro). (TIF) [file pone.0054503.s002.tif]

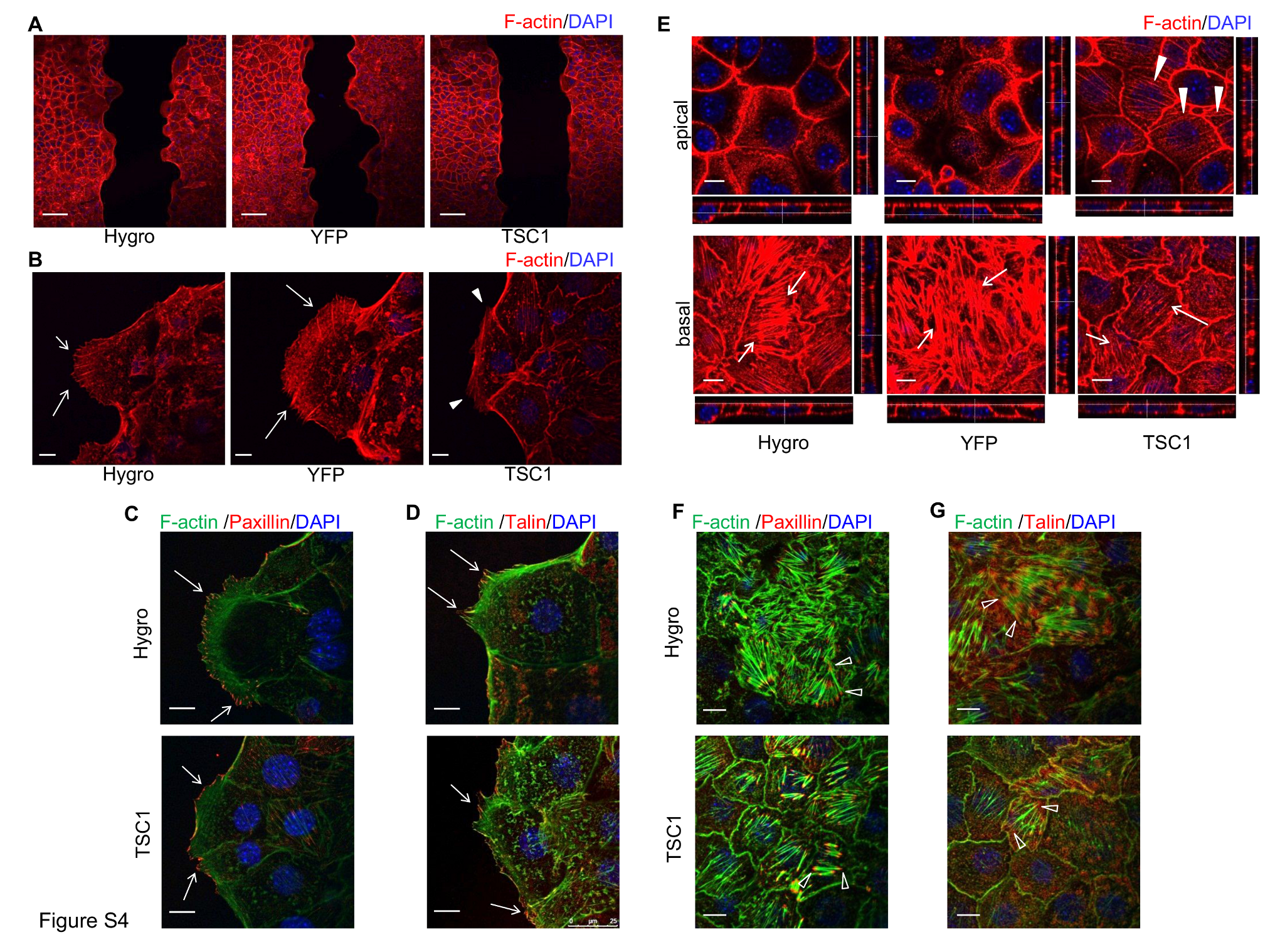

Supplement: Figure S4 — TSC1 alters actin cytoskeleton not only in the basolateral but also the apical region in cells of another independent clone set. (A) Low-magnification confocal images of phalloidin stained cells. Scale bars: 100 µm. (B) High-magnification images of the leading edge. Representative images are shown. In each panel, left side is the scratch edge. Arrows indicate the actin fibers in the filopodia of CACL1-Hygro cells and -YFP cells. Arrowheads showed actin fibers in the filopodia of CACL1–TSC1 cells. Scale bars: 10 µm. (C, D) Scratched cells were stained for F-actin and components of focal adhesion. Representative merged images of F-actin and paxillin (C) or talin (D). Focal complexes appear as small dot-like structures (arrows). Scale bars: 10 µm. (E) TSC1 reduced basal actin fibers and induced apical actin fibers. Images show representative X-Y sections from scans at 0.5 µm steps from the basal (close to the substrate, lower panels) to the apical side (upper panels) of the cell. X-Z (top to bottom) and Y-Z projections (left to right) are shown at the bottom and right side of each panel, respectively. Dotted line indicates the level of X-Y images shown. Arrows denote actin stress fibers in the basal side of cells. Arrowheads indicate the actin fiber network in the apical side of CACL1-TSC1-11 cells. Scale bars: 10 µm. (F, G) TSC1 inhibited formation of focal adhesions in the confluent stage. Cells in confluent monolayer were stained for F-actin and paxillin (F) or talin (G). Open arrowheads show focal adhesions connected to stress fibers. Scale bars: 10 µm. (TIF) [file pone.0054503.s004.tif]
